# *Carmenta theobromae* (Busck, 1910), pest of guava in Colombia: biology, life cycle and natural enemies

**DOI:** 10.1016/j.heliyon.2020.e05489

**Published:** 2020-11-13

**Authors:** Victor Camilo Pulido-Blanco, Orlando Ildefonso Insuasty-Burbano, Zaida Xiomara Sarmiento-Naizaque, Julio Ramírez-Durán

**Affiliations:** a Corporación Colombiana de Investigación Agropecuaria (Agrosavia), Centro de Investigación Tibaitatá, Sede Cimpa, Santander, Colombia; b Faculty of Sciences, Universidad Pedagógica y Tecnológica de Colombia, Colombia

**Keywords:** Agronomy, Biological pest control, Biological sciences, Ecology, Anatomy, Entomology, Insects, *Psidium guajava*, Integrated pest management, Life cycle, Bander worm, *Carmenta theobromae*

## Abstract

In 2006, the presence of a pest in guava was detected for the first time in the Province of Vélez, Santander, Colombia, known as the bander worm. Research on the biology of this pest is scarce and no natural enemies have been registered. The aim of the study was to establish the taxonomy, life cycle, damage (distribution, incidence, and severity) and natural enemies of this pest to be used in future integrated management programs. This study was carried out between May 2013 and December 2014. The taxonomy and morphological descriptions of the life stages of the bander worm correspond to *Carmenta theobromae* ( Busck, 1910). The life cycle in the field was 120–150 days, with 2–3 generations per year: egg, 15–30 days; larva, 60 days; pupa, 25 days; adult, 10–30 days. In the laboratory, the life cycle was 90–110 days: egg, 10–20 days; larval stages 6–7, 50–60 days; pupa, 20–22 days; adult, 5–7 days. The incidence was 98% in 124 farms with 9.87 ± 1.94 infested trees in relation to 40.74 ± 5.52 observed trees (*n* = 4,970). Severity was moderate (*n* = 48). The damage involves the removal of the bark to reach the vascular cambium. Biological control associated with the parasitoids *Brachymeria pedalis* and *Telenomus* sp., the entomopathogens *Lecanicillium lecanii*, *Beauveria bassiana* and *B. brongniartii*, and the practices like weeding and pruning represent a potential control strategy.

## 1 Introduction

In Colombia, guava (*Psidium guajava* L.) is produced mainly in small farming units within silvopastoral systems of less than 2 ha, at altitudes ranging from the sea level to 1,900 m a.s.l (Morales and Melgarejo, 2010). Although guava stands out due to its socioeconomic importance, the distinct technological gap affects market competitiveness (Insuasty et al., 2007). The province of Vélez and the northern region of the department of Valle del Cauca in Colombia contributes with 49% of the national guava production (239,713 t/year) with 33% (CAGI, 2013) and 16% (Carrillo and Ocampo, 2011), respectively. However, the presence of pests and diseases affects productivity in guava orchards (CAGI, 2013).

**Figure 1:**
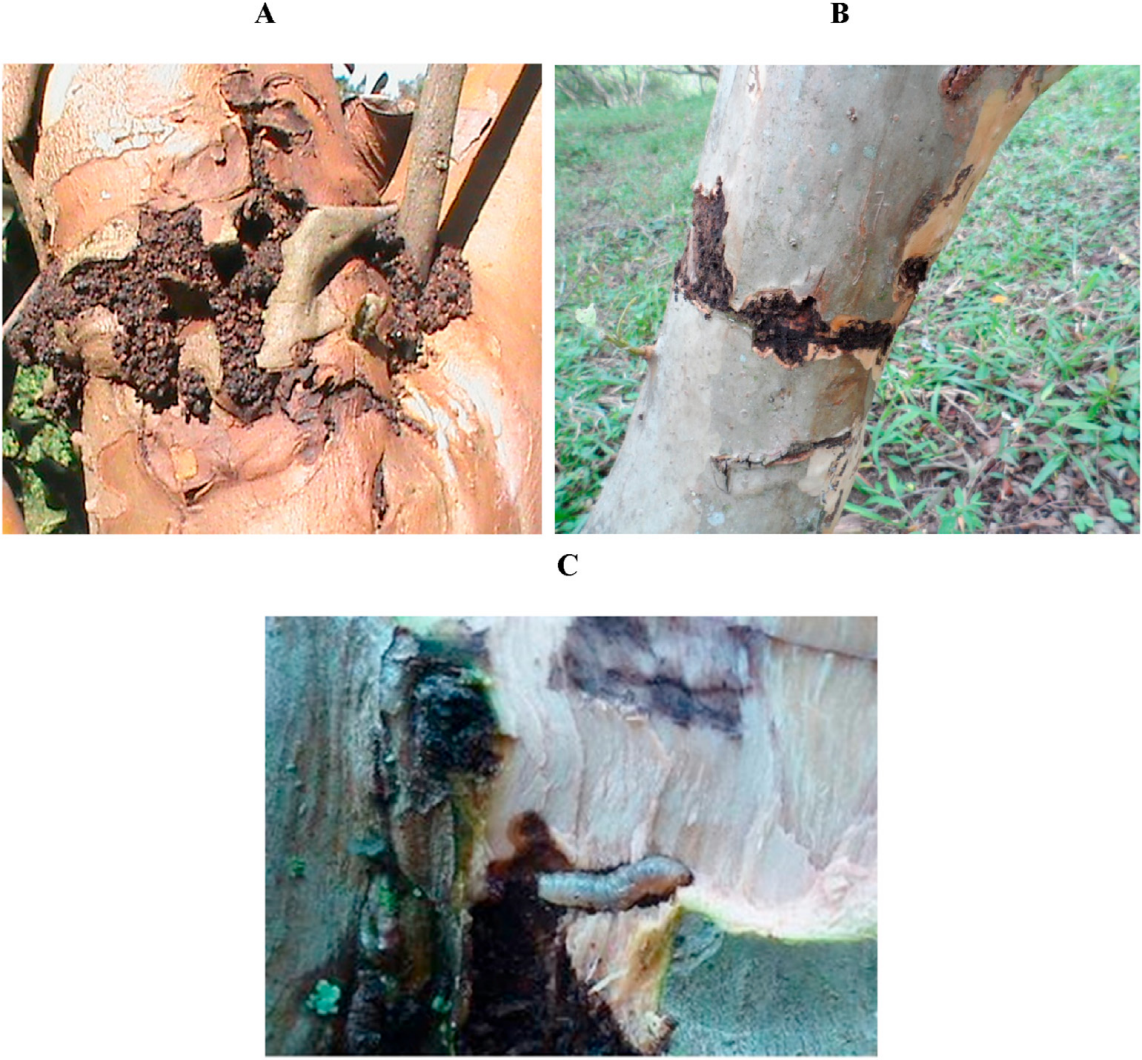
External appearance of the habitat of the “bander worm” A. Drilling around the stem. B. Typical scar in form of a ring. C. Larvae and the stem lesion caused by the bander worm.

Within the pest complex found in guava orchards in the province of Vélez, the guava fruit fly *Anastrepha striata* (Schiner, 1868) and the guava weevil *Conotrachelus psidii* (Marshall, 1922) (Instituto Colombiano Agropecuario ICA, 2012) are of great importance. Recently, a stem borer known as “gusano anillador” in Spanish or “bander worm” has been the subject of concern in this guava producer region; in addition, in the northern region of the department of Valle del Cauca, this plague has also been detected in cacao tree plantations. In the province of Vélez, the bander worm has been found feeding below the bark of guava trees until it reaches the vascular cambium forming rings around the stem (Figure 1) (Pulido Blanco, 2013).

Xylophagous species are typically not dependent on environmental humidity, a mechanism that favors them even in times of drought (Bakowski et al., 2010). Yet, aspects of its biology and control mechanisms are unknown. Therefore, the aim of the study was to establish the taxonomy, life cycle, damage (distribution, incidence, and severity) and natural enemies of this pest to be used in future integrated management programs.

## 2 Materials and methods

### 2.1 Study site

The experimental work was carried out in plots located in the province of Vélez, department of Santander, Colombia, in the municipalities of Vélez, Jesús María, Guavatá, Puente Nacional, San Benito, and Barbosa. Additional plots were studied in the municipality of Moniquirá, in the province of Ricaurte and in the municipalities of Briceño and Tununguá in the western region of the department of Boyacá. Laboratory studies were carried out in the agricultural entomology laboratory of C.I. Tibaitatá, sede Cimpa, ascribed headquarters to Corporación colombiana de investigación agropecuaria (Agrosavia), and located at 5° 56′ 51″ N and -73° 36′ 24″ W.

### 2.2 Taxonomic identification

To establish the family of this pest, the lepidopteran code of the Taxonomy Research and Information Network of the Government of Australia was used in its online version (CSIRO, 2013). Descriptions of the adults of the “bander worm” and subsequent taxonomic research to establish the possible genus were corroborated with the specialists: Nereida Delgado Puchi of Venezuela, Jorge L. Pinto of Colombia and Patricia Gentili-Poole of the Smithsonian Institute of Panama. For the identification of the species, comparisons were made with the original external description of *C. theobromae* (syn. *Sesia theobromae*) by Busck (1910), as well as with the works of Delgado (2005) and Sarmiento-Naizaque et al. (2016). A male and female were dissected to extract the genitalia through the Hanneman (1957) and Hardwick (1950) protocol. The characters of genitalia were compared with those described by Morán-Rosillo and Castillo-Carrillo (2020) and the photographs were sent to specialist Gilson Moreira in Brazil. The specimens were deposited in the “Colección Taxonómica Nacional de Insectos Luis María Murillo” (CTNI), Mosquera, Cundinamarca, Colombia.

### 2.3 Lifecycle

The lifecycle of the pest was studied in the field and laboratory. In the field, weekly inspections of pest damage were carried out in guava trees in the province of Vélez, between July 4, 2013 and December 24, 2014. The frequency of collection of the egg, larva, and pupa stages was recorded, collected by direct capture, and adults, collected with entomological net.

Eggs, larvae, pupae and adults were collected in farms and then studied under laboratory conditions, emulating field conditions, i.e. with a temperature of 25.4 ± 4.93 °C, relative humidity of 55.6 ± 11.58%, and a photoperiod of 12: 12 h (light: dark). Conditions were constantly monitored using the MicroLogPRO II® datalogger.

A sample of 96 eggs was examined, which were isolated and placed in Petri dishes. Morphology, color (Munsell color tables) and size (width-height) data were recorded using a stereoscope and a microscope with a micrometric 1: 100 mm ratio at 4X and 10X magnification (Pulido-Blanco et al., 2019). The number of hatches and non-viable eggs were checked daily, according to the method of Sarmiento-Naizaque et al. (2016). Differences between egg length and width were analyzed with a Student's t-test at 5% significance as mentioned by Delgado (2005).

The larvae collected (*n* = 359) were measured using an entomological vernier to register and establish ranges by size (Pulido-Blanco et al., 2019). The intervals were established with a confidence level of 95%, corresponding to insects of small (5.1 ± 0.47 mm in length, α = 0.05, *n* = 50), medium (10.63 ± 0.31 mm long, α = 0.05, *n* = 76) and large sizes (14.29 ± 0.25 mm long, α = 0.05, *n* = 70). Each larva was placed in a transparent plastic container with a specific diet. The cultures used were an artificial diet proposed by Garcia and Haro (1986) (*n* = 56 larvae) that included fresh guava tree sawdust (*n* = 250 larvae) and a semi-artificial diet elaborated in the Biological Control Laboratory of Agrosavia, CBB for *Spodoptera frugiperda* (Smith, 1797) (CORPOICA, 2013a, CORPOICA, 2013b) with guava sawdust (*n* = 53 larvae). Sarmiento-Naizaque et al. (2016) used this last diet in order to study the number of larval stages. For this, each larva was placed inside a plastic container with 5 cm^3^ of semi-synthetic diet.

Larvae were checked daily for cephalic capsules and the distance between genae was measured. Data were analyzed with the Dyar coefficient published by Parra and Lara (1989) contrasting the results with the number of molts observed.

The pupae (*n* = 300) collected and obtained in the laboratory were kept in plastic cups containing cotton moistened with sterile water; the containers were sealed with tulle fabric and fastened with elastic bands. Dead pupae were kept in a glass container with a butyl stopper and alcohol at 70% v/v (Pulido-Blanco et al., 2019).

The adults that emerged in the laboratory were placed in entomological cages in a 5: 5 male to female ratio, 30 pairs in total, accompanied by a drinker, rehydrated guava flour, cotton impregnated with 10% sugar solution, another cotton impregnated with honey water at 10%, and 2 g of multiflora pollen (Pulido-Blanco et al., 2019). Several paper napkins were placed on the walls of the cage to collect the egg masses and quantify the number of eggs (Pulido-Blanco et al., 2019). Dead adults were conserved in the entomology laboratory of Cimpa and photographs of specimens were sent to specialists to corroborate the taxonomic identification.

An evaluation and description of the life stages were made every two days, recording the state and ethology observed. Duration (days) of each of the life stages was recorded, under a statistical design with measurements repeated over time and comparison of means with Student's t-test for the average of each state between the cohorts (if any), at a range of 95% confidence. Atypical data were rejected using the Grubbs test at 95% confidence.

### 2.4 Damage description

In order to recognize and describe the damage and its evolution, four farms with the presence of the “bander worm” were selected. Weekly follow-ups for a period between May 2013 and December 2014 were carried out in agreement with the owners. Following the methodology described in Pulido-Blanco et al. (2019), in total 72 trees were randomly assigned to four farms (two in the municipality of Moniquirá and two in Vélez); of these trees, 48 were of interest for the evaluation of the “bander worm” damage and 12 individuals were characterized per farm. Of these, six trees were infested with the “bander worm” and six were “healthy trees” without the presence of the pest (but that could be affected by other pests naturally present in the field). Further, each tree was labeled with a consecutive number (Pulido-Blanco et al., 2019).

Weekly inspections of injury presence caused by the pest, number and condition of these (fresh: recent damage; outdated: inactive damage) and of the symptoms, were carried out in each tree. All lesions were marked with white ink (liquid paper corrector) to facilitate follow-up observations and description of their evolution. Data were registered based on a severity scale with six defined categories for the pest proposed in this study based on observations of the health status of trees attacked in the field (Table 1). During each inspection temperature records (maximum and minimum), relative humidity (%) and rainfall (mm) were taken at each of the monitoring farms (Pulido-Blanco et al., 2019). Likewise, and throughout the methodological phase, photographic records were taken.

**Table 1:** Proposed damage scale to monitor the severity of the pests in guava fields.

Degree	Symptom	Bander worm
0	Absent	Absent
1	Very mild	Outdated (old lesions)
2	Mild	1 -2 active lesions
3	Moderate	3–4 actives lesions
4	Severe	5–6 actives lesions
5	Very severe	≥7 actives lesions

### 2.5 Natural enemies

We identified natural enemies from eggs, larvae, and pupae collected in productive sector farms. All parasitoids found from these immature stages were conserved in alcohol at 40% v/v for their taxonomic identification. The number of affected stages per morphospecies was registered, as well as the type of parasitism, the stage in which the pest was attacked, and the number of parasitoid individuals per attack (Pulido-Blanco et al., 2019). Some specimens were sent to taxon specialists for identification. The two most frequent parasitoids that attack various stages of the pest's life cycle are privileged over others, as an input for a future integrated management program.

Entomopathogenic fungi were isolated from larvae and pupae found in samples brought from the field and obtained under laboratory conditions following the methodology described in Pulido-Blanco et al. (2019): scraping of mycelium from the surface of the infected live larva or pupa, and then planted in Potato Dextrose Agar (PDA). The cultures were maintained at 28 °C and 80% relative humidity for seven days, after which they were partitioned in PDA microcultures under the same conditions. Once the macroscopic fruiting bodies were identified, direct observations were made in fresh at 40X with an Olympus BX40 fluorescence microscope for reproductive structure recognition (Pulido-Blanco et al., 2019). Microcultures were identified by the pathogen identification service of the Laboratory of Biological Control, Faculty of Agronomy, Pedagogical and Technological University of Colombia (U.P.T.C.).

At the same time, the chemical agents, presentations, and brands more commonly used by growers and collectors of the guava producing agroecosystem, or *Guayabero* in Spanish, of the municipalities considered in Santander and Boyacá were identified through a survey carried out with fruit producers and collectors.

## 3 Results and discussion

### 3.1 Taxonomic identification

The specimens were cataloged under the number 6245, with the following data: *Carmenta theobromae*; Sesiidae; Lepidoptera; det: Zaida Sarmiento y Víctor Pulido, 2020. Colombia; Santander; Barbosa; Fca. El Hogar, 1575 m; N 5°56′51″, W 73°36′24″; 2020. J. Jiménez; xilófago de *Psidium guajava* (Myrtaceae)-guayaba. Other previously collected specimens of the same species was catalogued under the number 3423, with the following data: Santander, Vélez, 2013. Det. Víctor Pulido. All specimens are in the “Colección Taxonómica Nacional de Insectos Luis María Murillo” (CTNI), Mosquera, Cundinamarca, Colombia.

Two possible genera for the guava stem bander worm were identified: *Synanthedon* sp., and *Carmenta* sp. After specialists corroborated the genus and the identity of the species was settled corresponding to *Carmenta theobromae* (Busck, 1910). This species was described by Busck (1910), as *Sesia theobromae*, from a male specimen emerged from a cocoa fruit collected on the island of Trinidad (Eichlin, 1995). In Colombia, Gallego and Veléz (1992) reported the presence of this species as a stem borer guava. The most recent report that is geographically closer to Colombia come from a study carried out with cacao seeds in the state of Aragua in Venezuela in 2005 (Delgado, 2005). Thus, it is thought that *C. theobromae* went from consuming cacao pods and seeds to guava tree stems, an argument that agrees with what has been reported by Harms and Aiello (1995). These authors consider that the species whose larvae are fruit drillers have an aberrant behavior or even completely different compared to most species of the Sesiidae family. In their larval stage, these individuals are characterized by preferring to perforate stems, branches or roots (Heppner, 1987).

**Figure 2:**
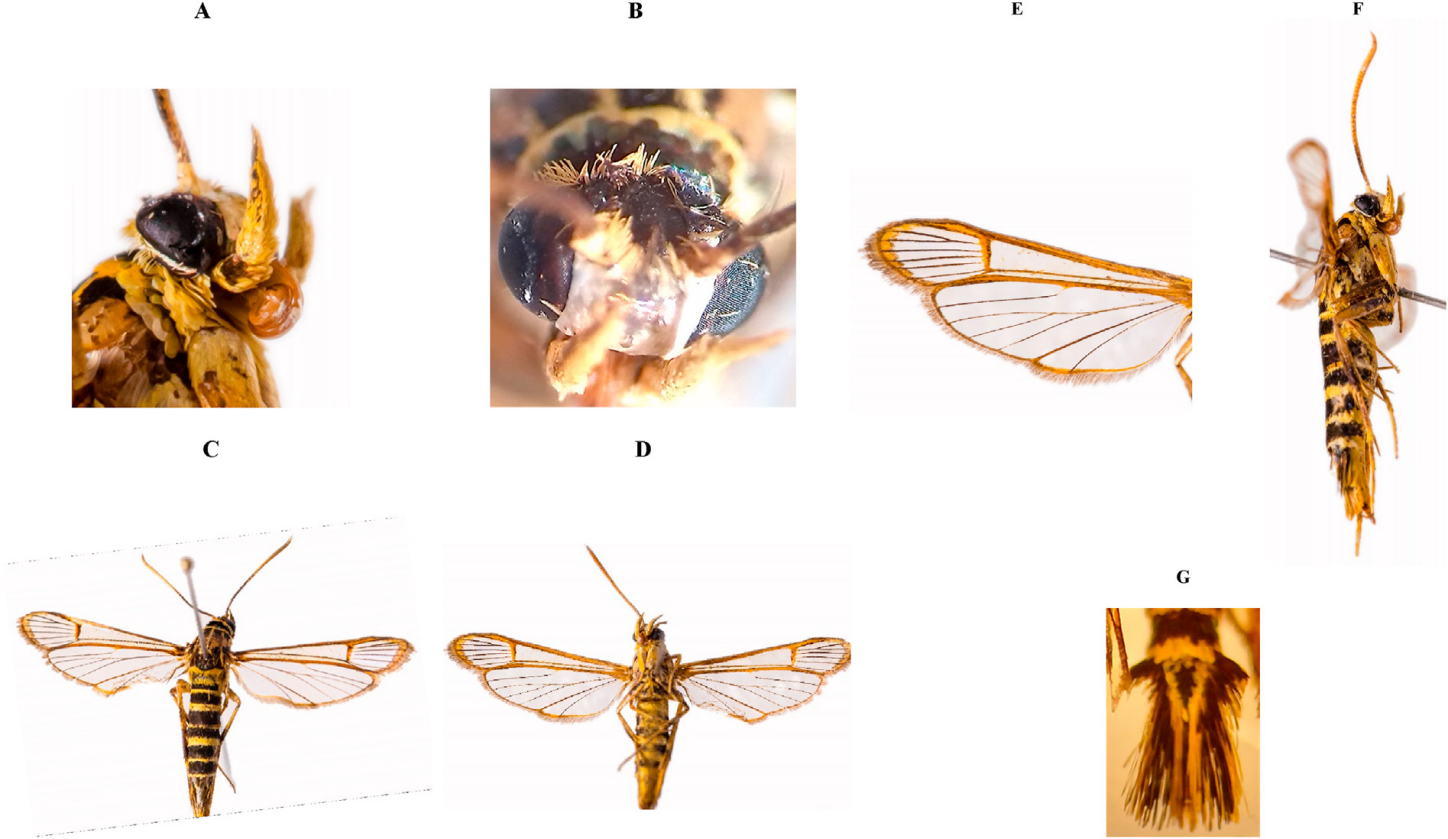
A. Labial palpi yellow. B. Silver face. C. Upper body black with yellow. D. Lower body yellow. E. Wings transparent, with a wide terminal edge of dark brown color and brown cilia. F. Legs bluish-black on the exterior surfaces. G. Large anal tuft, paddle-shaped.

The morphological characteristics of the life stages of bander worm coincide with those reported by Delgado (2005) and Sarmiento-Naizaque et al. (2016). As characters of the adults that corroborate the identity of the species are the bright yellow labial palps (Figure 2A), face silvery with a blue-black triangle and the yellow of the vertex (Figure 2B), black head (Figure 2B), the black antennae with ochreous underside Figure 2C, D, F), yellow collar (Figure 2A), thorax with three yellow longitudinal lines and a posterior fringe of yellow hairs (Figure 2C), transparent and iridescent wings with deep purplish black veins and rib border and a fairly wide terminal border of dark brown color (Figure 2E); cilia blackish brown (Figure 2E). Abdomen black, each segment with a yellow transverse band, all of them narrow except for the middle, which is twice as wide as the others (Figure 2C). Underside of body yellow (Figure 2D). Legs blue-black on the outer surfaces (Figure 2F), yellow towards the body (Figure 2D) Large, paddle-shaped anal tuft, black with some yellow lateral scales (Figure 2G) (Busck, 1910) (see Figure 2).

**Figure 3:**
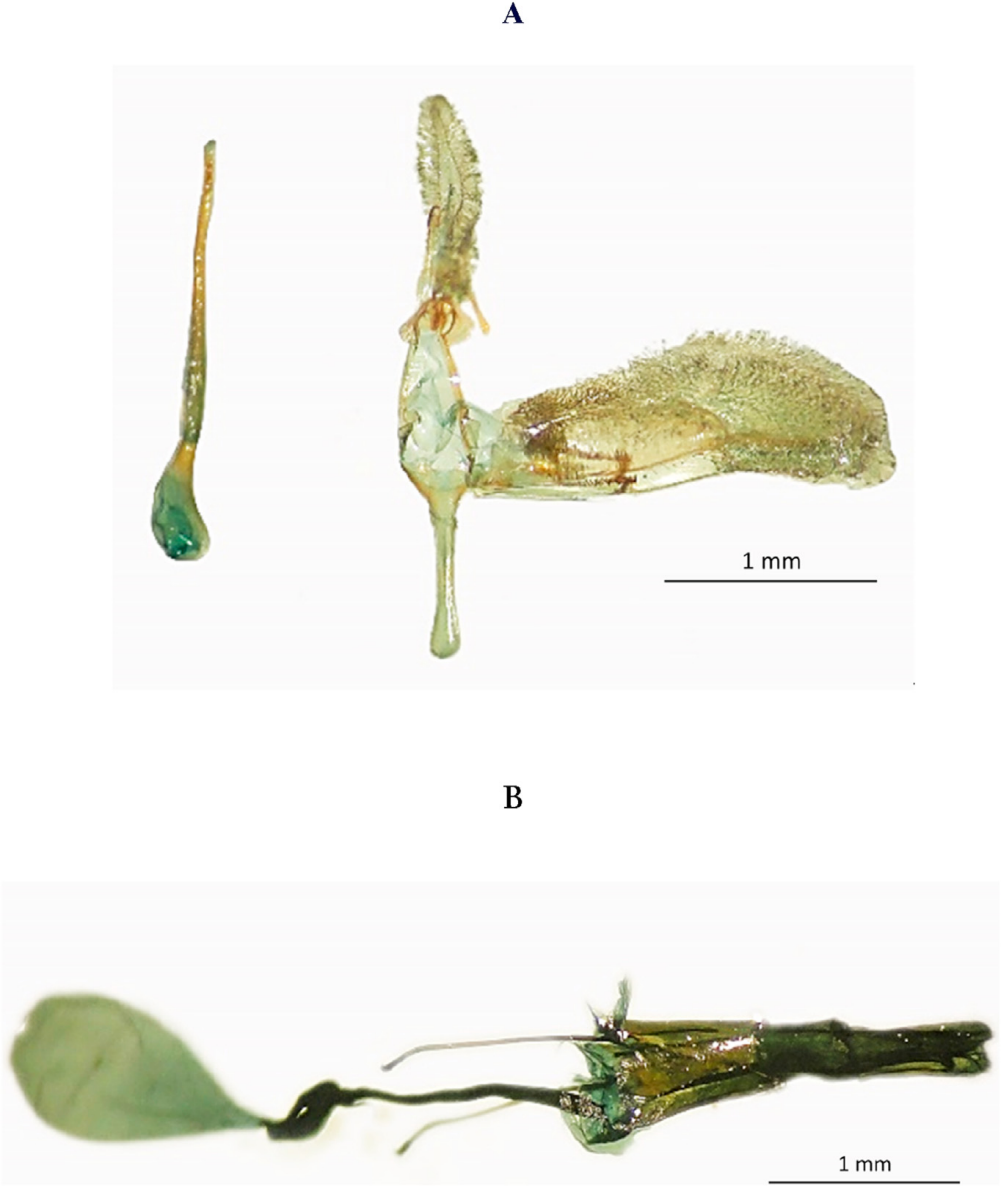
*Carmenta theobromae* genitalia A. Male genitalia. B. Female genitalia. Photographs: P. Osorio (2020).

The characteristics of the genitalia adjust to *C. theobromae* as described in Moran-Rosillo and Castillo-Carrillo (2020) (Figure 3). In the male genitalia, the valvas are elongate and have laminar hairs like uncus. In the female genitalia, the antrum is slender, somewhat sclerotized and pigmented, the ductus bursae is broader than antrum.

### 3.2 Lifecycle

In the field, 61 consecutive registries were made from July 4, 2013, to December 24, 2014, finding that the lifecycle of the bander worm under natural conditions is 120–150 days (4–5 months), with two to three generations in one year (multivoltine). In the egg stage it lasts 15–30 days (1/2 to 1 month); larva about 60 days (2 months); pupa approximately 25 days (5/6 months) and adults from 10 to 30 days (1/3 to 1 month). Of the total number of eggs found in the field, 75.6% had already hatched and only 23.7% were found without hatching. It is worth noting that egg, larva and pupa stages share the same microhabitat inside the guava tree.

**Figure 4:**
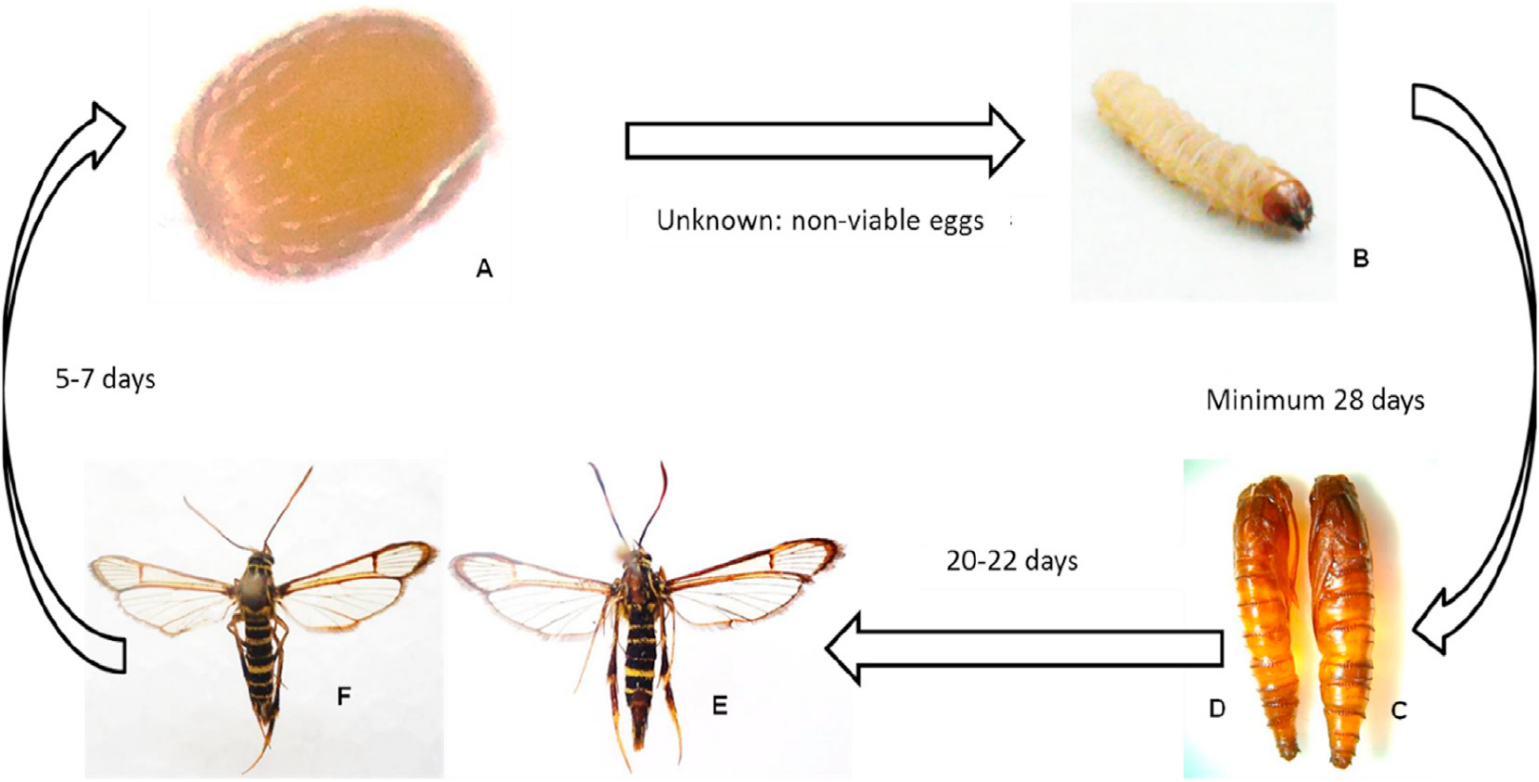
The lifecycle of the guava bander worm.A. Egg. B. Larva. C. Pupa ♀. D. Pupa ♂. E. Adult ♀. F. Adult ♂.

Under laboratory conditions, more than 500 entries were registered corresponding to laboratory monitoring of more than 120 specimens of the bander worm. Data were registered during 18 months of monitoring. The lifecycle of *C. theobromae* under laboratory conditions mentioned in the Material and methods section is as follows: unknown egg (no fertile eggs were obtained); larva, 6 to 7 instars, 50–60 days; pupa, 20–22 days; and adult, 5–7 days (Figure 4). In this study, adults that lasted 14 days were registered. In general, the lifecycle was shorter than what had been recorded in the field, with a range of 90–110 days (Pulido Blanco, 2014).

By estimating the Dyar coefficient, Sarmiento-Naizaque et al. (2016) obtained a direct relation of 87.7% between the capsule width and four possible stages under laboratory conditions. However, Cordo et al. (1995) reported in different Sesiidae species up to seven molts, which would indicate that in their natural environment, larvae could even have three more molts.

From the observations recorded for each life stage of this pest, the fact that the larvae of the bander worm feed in a similar way to what was observed in their natural conditions, with a tendency to drill in a circular direction, can be highlighted. Nonetheless, egg masses obtained in the laboratory from adults placed in entomological cages were not viable.

### 3.3 Damage description

**Figure 5:**
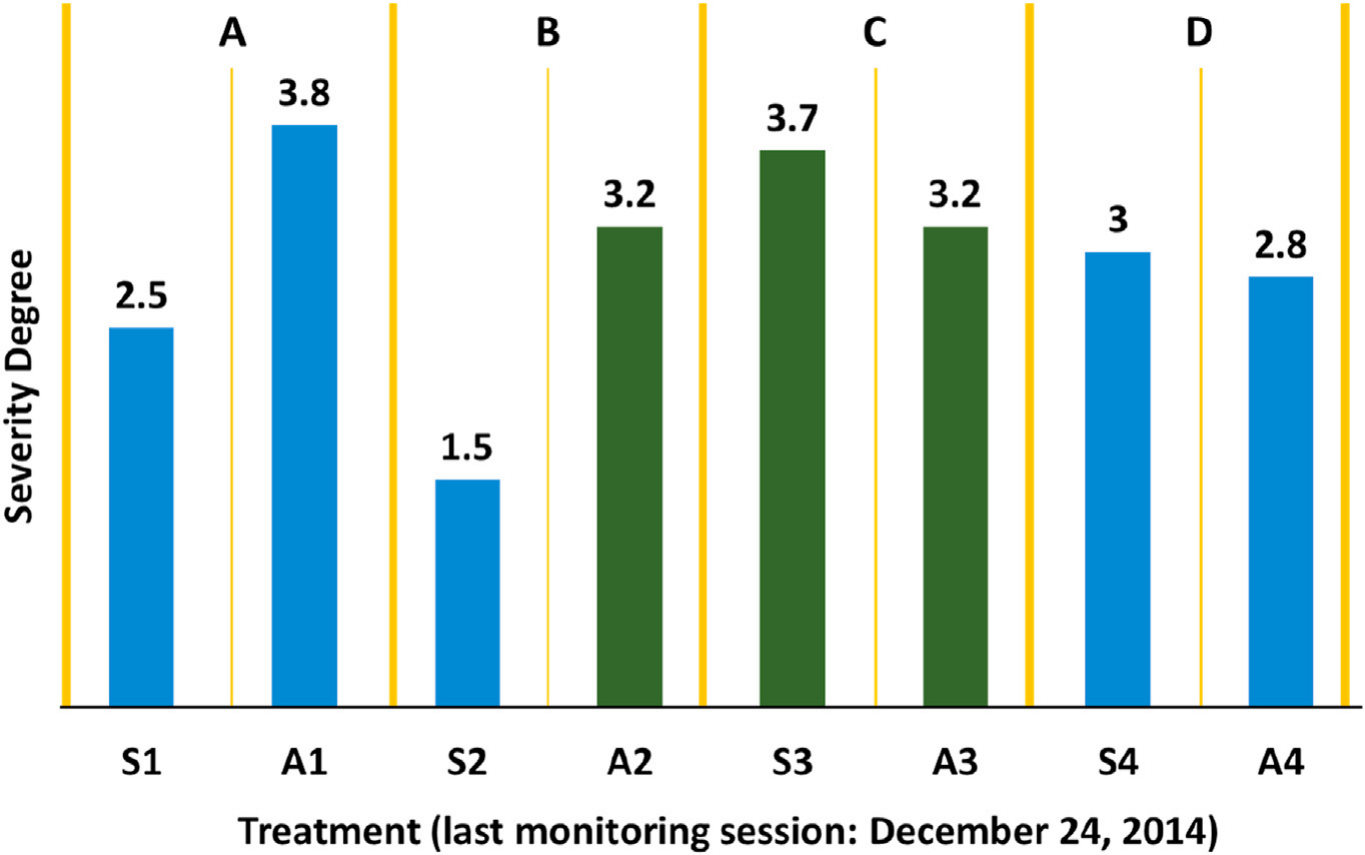
Average degree of severity registered for *C. theobromae* in evaluation farms after 518 days of monitoring. A. Farm 1 - Moniquirá (Finca Villa Mónica, Rosendo Pineda). B. Farm 2 - Moniquirá (El Triunfo, Carlos Motta). C. Farm 3 - Vélez (El Paraíso, Rosalba Ramírez). D. Farm 4 - Vélez (El Pomorosal, Carlos Vargas). S: “Healthy trees”; A: trees affected by the bander worm.

In 61 follow-up weeks, 4,392 records were carried out in the assessment of 72 guava trees. After 518 days of follow-up, a mean severity of moderate was obtained (Figure 5) in the four evaluation sites considered; in addition, the mode was severe (4).

**Figure 6:**
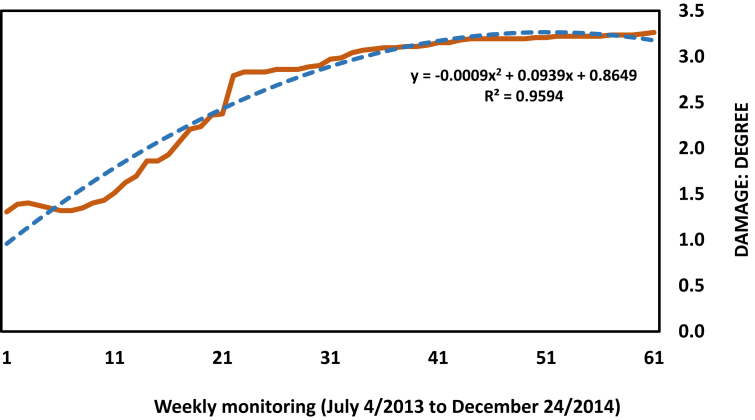
The trend of cumulative damage (degree) during 61 weeks of monitoring in four farms.

For sites 1 and 2 in Moniquirá a mode of 3 (almost 4) was registered; for sites 3 and 4 in Vélez a mode of 5 was recorded. Compared with the data of the immediately preceding year, a sustained tendency of damage increase caused by the bander worm is clear (Figure 6); this indicates the establishment of the populations in the guava trees inspected.

**Figure 7:**
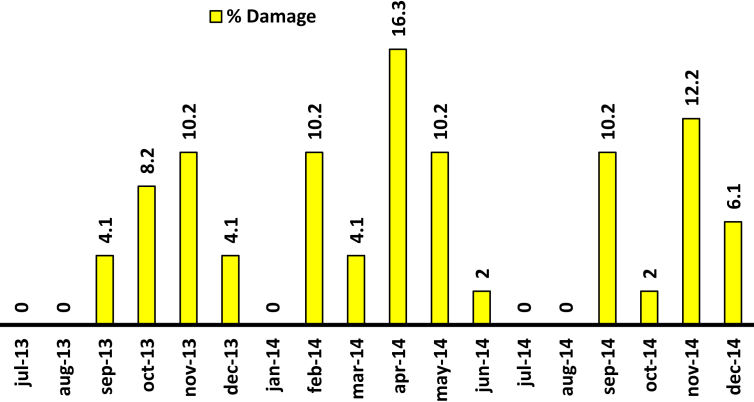
Dynamics of monthly damage caused by the larva of *C. theobromae* during 17 months of evaluation in the province of Vélez.

Regarding the damage evolution over time, two very pronounced peaks of larval activity occur within a year, one in the months of October–November, and the highest peak in the months of April–May. There are two periods without activity registered between July–August and at the end of December–January (Figure 7).

**Figure 8:**
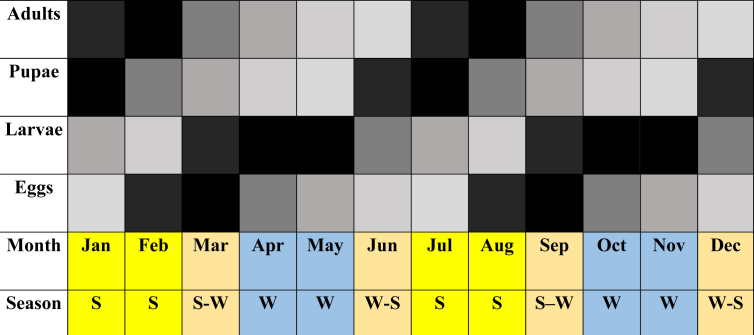
Frequency of capture of the life cycle stages of *C. theobromae* in the field. The higher the intensity of the color, the higher the frequency of capture. S: Summer; W: Winter. The relative collection rate is specified for the larval stage collected per tree:  Zero to five individuals;  six to ten individuals;  eleven to fifteen individuals;  Sixteen to twenty individuals;  twenty one to thirty individuals;  more than thirty-one (up to 96) individuals.

These data were matched with the bimodal rain cycles of the equatorial tropics (Figure 8), indicating that the bander worm shows several generations throughout the year with overlapping states.

The overall picture of the damage caused by the bander worm was analyzed using the female of *C. theobromae*. Once the female has identified a tree with the right colonization conditions, she deposits the eggs in open spaces found in the bark with access to fresh vascular cambium tissue. Under laboratory and field conditions, oviposition is carried out in the morning periods from 10:00 to 12:00 h, and from 16:00 to 18:00 h in the afternoon. Egg laying occurred under the bark, sheltered inside a stable microhabitat in an independent environment (with a temperature that is 5 °C below the environmental temperature, and humidity with 10 percentage points above the relative humidity observed in the environment).

When larvae hatch, they have access to the site where they thrive. After a period of 15 days of feeding below the bark, the first vestiges of activity begin to be observed, i.e. a bulge in the bark of approximately 2 cm in length. Most of the times the bulge was observed running horizontally. Larvae pass all its stages under the bark (seven stages at 25 °C and 70% of relative humidity) while devouring the internal cortex, the phloem, and the primary xylem until reaching the vascular cambium.

Larvae were not observed penetrating beyond the sapwood or secondary xylem, sticking only to the ring of the tree. Approximately 40–50 days later, the wound extends 15–25 cm, and pupae are found from where the adults will emerge. From this point, wounds can reach a width of 4–20 cm, depending on the number of larvae that cohabit the damaged area. In the field, 2 to 40 larvae have been found in a single wound.

The most typical feature of the wound is the accumulation of larval excreta. These are irregular spheres of 2 mm in diameter that the larvae use to seek shelter and hide from predators, as well as being used as material for cocoon manufacturing. Finally, the wounds are abandoned; however, they can become re-infected by another generation. It should be noted that the same tree could simultaneously have outdated (old) and active damages on the stem, axillae, branches, and apices of cut branches. Up to 14 simultaneous wounds in an individual tree were found.

The bander worm devours the fresh tissue of the tree represented by the phloem, xylem and vascular cambium, in such a way that it affects the nutrient and water transport systems of the guava tree, as well as its growth. Therefore, the symptoms associated with the damage done by the bander worm corresponds to those typically observed in photosynthates, elements and water translocation, i.e. yellowing (chlorosis) and reddening of leaves, epinastia (leaf fall), loss of vigor, sprouts below the damaged area, and induction of calluses circumscribed to the wound.

In young plants and trees not reaching five years of age, atrophy and even death might occur. However, trees older than five years withstand the damage. Further, trees from 10 years of age and older do not show symptoms. In this sense, the pest acts as a good parasite because it does not kill its host.

### 3.4 Natural enemies

Eighteen (18) species of parasitoids and five (5) species of entomopathogens were found (Burks, 1960; Townes and Townes, 1966; Masner, 1976; Quicke and Sharkey, 1989; Whitfield, 1997; Campos, 2001; Gauld et al., 2002; Sharkey, 2004; Fernández and Sharkey, 2006; Campo-Moreno, 2007; Valerio et al., 2009; Johansen, 2010). Regarding the first, 318 were Hymenoptera and four (4) were Diptera; and of the latter, we found 34 events of fungi over bander worm stages collected in the field and studied in the laboratory (Table 2). The most abundant family was Braconidae with 7 genera. The most frequent parasitoids per case were *Brachymeria pedalis* (Cresson, 1872) and *B. conica* (Ashmead, 1904), with 40% of bander worm pupae parasitized (240 cases). Of these two, *B. pedalis* was more conspicuous, prevailing in 70% of cases. The most frequent parasitoids by number were *Apanteles* sp, with 232 individuals. The two most frequent types of parasitism were solitary pupae parasitism and koinobiont larvae superparasitism, respectively.

**Table 2:** Biocontrollers identified in eggs, larvae and pupae stages of the guava bander worm.

Affected state	Biocontroller					
	Parasitoid	Picture parasitoid	#	Entomopathogen	Picture entomopathogen	**#**
Egg	*-Gryon*		1			
	*-Telenomus*		1			
Larva	*-Apanteles*		233	*-Beauveria bassiana*		2
	*-Bassus brullei*		1	*-Beauveria brongniartii*		1
	*-Bracon*		3	*-Paecylomyces* sp.		2
	*-Ganodes*		1	*-Paecylomyces lilacinus*		1
	*-Parapanteles*		7			
	*-Pimpla sanguinipes*		5			
	*-Scolomus*		4			
Pupa	*-Brachymeria conica*		7			
	*-Brachymeria pedalis*		4			
Larva and pupa	*-Baryscapus* cf.		32	*-Lecanicillium lecanii*		28
	*-Eurytoma*		1			
	*-Lissonota*		7			
	*-Polstonia*		6			
	*-Siphostumia*		1			
	*-Sturmiomima*		3			
	*-Toechorychus*		2			

#: number of individuals found.

Referring chemicals used to manage pests of guava, most farmers employ pyrethroids in 90% of the cases, and organophosphates (Malathion® 50) in 10% of the situations. Currently, the use of an insecticide called Success® 48 based on Spinosad is commonly employed, and its use is now widespread across the guava producing regions.

On the other hand, considering the bioassay carried out on the cultivation system and the incidence of pests, 1,368 insects were recorded on 342 trees for four months where follow-up registries were carried out. *C. theobromae* affects technically managed guava systems differently, versus non-managed or silvopastoral systems: we found that the pest can affect all the trees of a silvopastoral system, compared with less than 5% of affectation in a technically managed system. In addition, within the same farm, if a lot of trees are technically managed, they will have, on average, between a third to half of the affectation that the neighbors without management. We believe that this fact is due to the fact that the management of guava trees creates adverse conditions for the eggs and larvae of the first stages of *C. theobromae*: a clean trunk, without weeds around, has more light input, less humidity and higher temperature, which makes it less attractive to the pest. Therefore, we agree with Pulido Blanco (2014) that weeding and pruning are recommended practices for the management of this pest.

Lastly, the use of the pheromone Z, Z. octadecadienol acetate (ODDA) has been reported, which is a synthetic male attractant and main component of the sexual pheromone emitted by the females of the genera *Carmenta* Edwards and *Synanthedon* Hübner (Nielsen and Balderston 1973; Eichlin 1992; Eichlin and Kinnee 2002; Delgado 2005). This method, combined with Wilkilson or McPhail traps, would contribute to reducing the number of males available for copulation, considering that the proportion of males observed in this study was one for every five females. It should be noted that this method alone does not constitute an efficient form of control (Navarro et al., 2004; Sánchez et al., 2011; González et al., 2012).

## 4 Conclusions

The bander worm of guava trees was identified as *Carmenta theobromae* (Busck, 1910). Under natural conditions, the lifecycle of the bander worm is 120–150 days (4–5 months), with two to three generations per year (multivoltine). Under laboratory conditions, the lifecycle was 90–110 days. *C. theobromae* is disseminated throughout the province of Vélez, and can affect all guava trees of the silvopastoral system, up to a severe degree. Potential approaches to control this pest may include the use of the parasitoids *Brachymeria pedalis* and *Telenomus* sp., the distribution of the entomopathogens *Lecanicillium lecanii*, *Beauveria bassiana* and *B. brongniartii*, and the weeding and pruning as practices for the management of this pest.

## Declarations

### Author contribution statement

V. Pulido-Blanco: Conceived and designed the experiments; Performed the experiments; Analyzed and interpreted the data; Contributed reagents, materials, analysis tools or data; Wrote the paper.

O. Insuasty-Burbano and J. Ramírez-Durán: Conceived and designed the experiments; Analyzed and interpreted the data; Contributed reagents, materials, analysis tools or data.

Z. Sarmiento-Naizaque: Performed the experiments; Analyzed and interpreted the data; Wrote the paper.

### Funding statement

This work was supported by Ministerio de Agricultura y Desarrollo Rural de Colombia (MADR) and Corporación Colombiana de Investigación Agropecuaria (Agrosavia) through the Public Funds Agreement 1828 of 2013.

### Declaration of interests statement

The authors declare no conflict of interest.

### Additional information

No additional information is available for this paper.
